# Quantitative profiling of capsaicin content in seven chili pepper cultivars using optimized HPLC

**DOI:** 10.3389/fpls.2026.1749488

**Published:** 2026-05-07

**Authors:** Pramod Pandey, Jiang Huo, Qi Zhang

**Affiliations:** 1 Department of Population Health and Reproduction, School of Veterinary Medicine, University of California, Davis, Davis, CA, United States; 2 Department of Environmental Toxicology, University of California, Davis, Davis, CA, United States; 3 Agricultural and Environmental Chemistry Graduate Group, University of California, Davis, Davis, CA, United States

**Keywords:** capsaicin, chili peppers, cultivars, extraction, HPLC, pungency

## Abstract

Capsaicin is the primary active compound responsible for the pungency of hot chilies (*Capsicum* spp.), functioning both as a deterrent to predators and as the trigger for the burning sensation produced by the activation of sensory neurons. It is also widely used in food and is associated with multiple health benefits for humans. This research focuses on quantifying and comparing capsaicin levels in various chili peppers to address the limited quantitative understanding of their pungency. The main objective was to determine capsaicin content across seven chili pepper cultivars (Habanero, Thai chili, Serrano, Jalapeño, Shishito, Poblano, and Anaheim) to better understand variations in spiciness. Using high-performance liquid chromatography (HPLC), the peppers were ranked from hottest to mildest based on capsaicin content. Results showed that Habanero had the highest capsaicin content (4,777 ± 588 mg/kg dry weight), while Anaheim had the lowest (0.9 ± 0.04 mg/kg dry weight). The overall ranking from hottest to mildest was: Habanero > Thai chili > Serrano > Jalapeño > Shishito > Poblano > Anaheim. Capsaicin levels in Habanero were considerably higher than in other varieties—158% higher than Thai chili, 357% higher than Serrano, and approximately 29,000% higher than Jalapeño. For quantitative determination of capsaicin, we used reversed-phase HPLC with UV-Vis detection. Notably, dry-pepper extractions yielded 542%–1,110% more capsaicin than wet-pepper extractions. This finding highlights the significant impact of drying on capsaicin extraction efficiency. Comparative analysis showed that hot chili varieties such as Thai chili, Habanero, and Serrano have very high capsaicin levels compared to Jalapeño, Shishito, Poblano, and Anaheim. HPLC-based quantification offers an objective and reproducible alternative to the Scoville Heat Units (SHU) scale for assessing the pungency of peppers. The findings of this study enhance understanding of capsaicin extraction and provide additional information into characterizing pepper spiciness, helping farmers, researchers, and the public gain deeper insights into the factors that determine pepper pungency.

## 1 Introduction

In hot chilies (*Capsicum* sp.), capsaicin is the compound responsible for their pungency, serving both to discourage predators and to elicit a burning sensation by activating sensory neurons. Beyond its sensory effects, capsaicin carries multiple beneficial biological properties that have prompted numerous investigations (Peplow, 2004; Whitfield, 2002). However, few studies compare capsaicinoids from more than five cultivars under standardized extraction conditions. For example, Kraikarun et al (Kraikruan et al., 2008a). evaluated only a Thai chili cultivar, highlighting the need for further studies to better understand capsaicin across various pepper cultivars. There is substantial interest in integrated omics approaches combining metabolomics, proteomics, and transcriptomics to better understand capsaicinoid (including capsaicin) biosynthesis (Bosland and Baral, 2007; Aza-González et al., 2011; Liu et al., 2023). Despite its therapeutic promise, the low natural yield of capsaicin and challenges associated with large-scale production continue to limit its broader industrial and clinical use. Thus, this study aimed to quantify and compare capsaicin levels in seven chili pepper cultivars (*Capsicum annuum*). The overall goal of this work was to determine capsaicin levels using HPLC and to characterize various chili peppers based on their capsaicin levels.

Currently, chili pepper is the most widely grown spice in the world, and it has many uses, including in cuisines, pharmaceuticals, and defense repellents (Bosland and Votava, 2012; Bosland and Baral, 2007). Globally, pepper cultivation is carried out on more than 3.68 Mha, producing annually 36.29 and 4.84 million tons of green and dry peppers, respectively (Kraikruan et al., 2008a). Compared to many other spices and crops, chili peppers are relatively easy to plant and grow under various climate conditions and are also easy to process. This may explain why chili pepper cultivation spread from America to Asia, Africa, and Europe (Kraikruan et al., 2008a; Liu et al., 2023).

**Figure 1:**
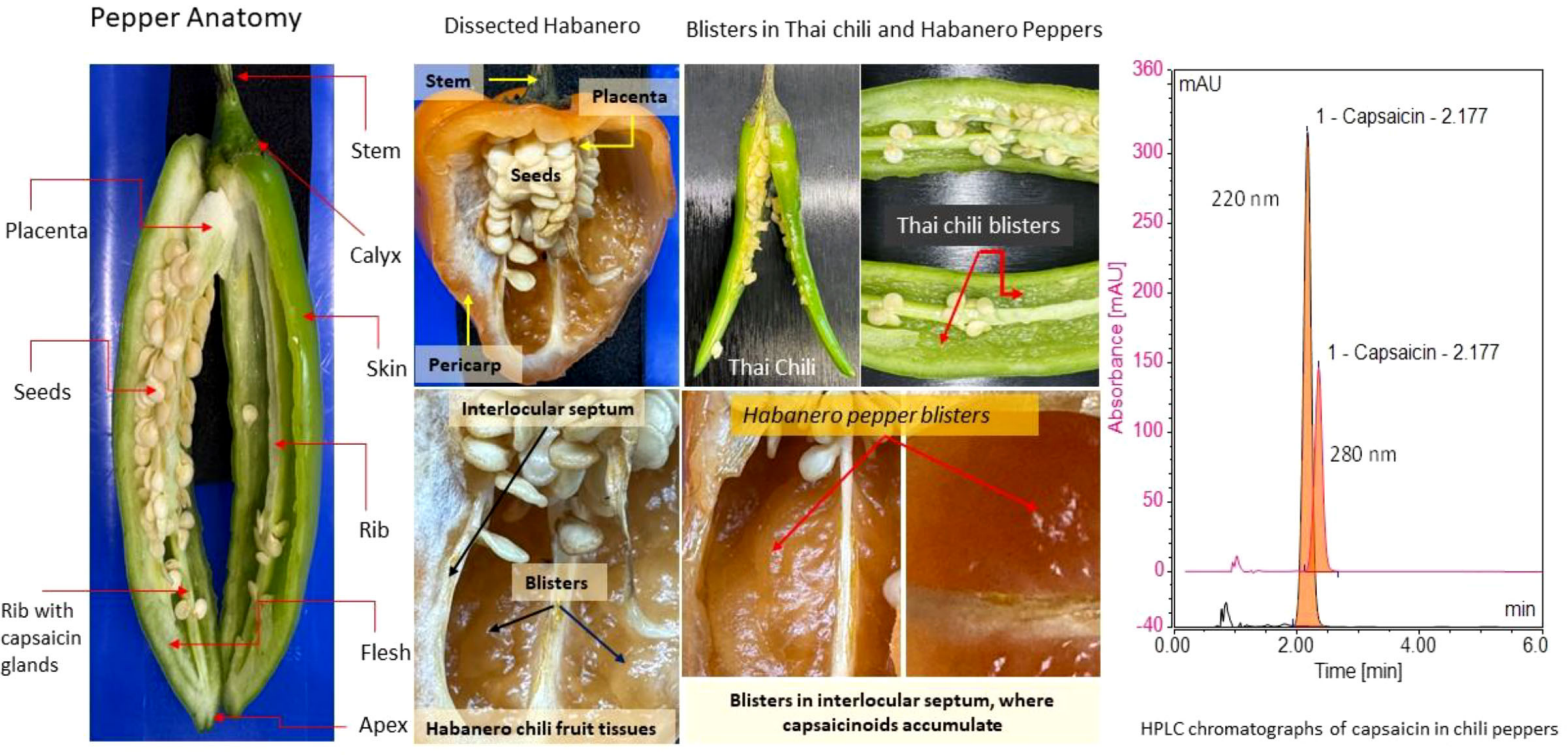
Chili pepper tissues, blisters, capsaicinoid vesicles, and capsaicin HPLC chromatogram.

In peppers, capsaicinoid accumulation starts approximately 20 days post-anthesis (i.e., after full flower opening) (Aza-González et al., 2011). Capsaicinoids are synthesized via biosynthetic pathways (phenylpropanoid and branched-chain fatty acid pathways) (Aza-González et al., 2011); however, the regulation of these pathways is not fully understood. A study by Bosland et al (Bosland et al., 2015). investigated “superhot” cultivars and found significant levels of capsaicinoids in pericarp tissue samples. In chili peppers that are not very hot, capsaicinoid-secreting vesicles are found only on the placenta. However, in superhot chilies, these vesicles are found in both the placenta and pericarp tissues, which contributes to their extreme spiciness (Aza-González et al., 2011; Bosland et al., 2015). The application of an advanced microscope, such as a stereo-fluorescence microscope, has illustrated that superhot chilies (Bhut Jolokia and Habanero) possess numerous capsaicinoid-containing vesicles throughout the pericarp tissues (**Figure 1**). In these superhot chilies, a large proportion of the pericarp tissue is covered with these vesicles, a feature that is absent in milder chili types (Bosland et al., 2015).

Moreover, only chili pepper fruits synthesize capsaicinoids, and accumulation varies depending on the type of chili pepper (i.e., a trait in chili peppers). The genetic makeup of chili peppers is critical for the biosynthesis and accumulation of capsaicinoids. For example, different cultivars show considerable variation in pungency and in the density of capsaicinoid-containing vesicles (Aza-González et al., 2011; Bosland et al., 2015). Aza-Gonzalez (Aza-González et al., 2011) showed numerous blisters (**Figure 1**) in Habanero peppers responsible for their spiciness. Analysis showed that the dissected Habanero tissue was filled with capsaicinoids in the interlocular septum, and these blisters were less common in Jalapeño (Aza-González et al., 2011; Bosland et al., 2015).

While capsaicinoids primarily evolved as a means of directed deterrence against herbivory (Tewksbury and Nabhan, 2001; Naves et al., 2019), they are also beneficial to human health. Previous studies have shown that many animals, including elephants, avoid chili plants (Whitfield, 2002), and that hot peppers repel pests mainly due to the capsaicinoids present (Peplow, 2004). Birds and reptiles, however, do not feel the heat of pepper. Birds, which are responsible for pepper seed dispersal, do not perceive capsaicinoids. Mammals are very sensitive to capsaicinoids and perceive pain and inflammation when capsaicinoids come into contact with their epidermal tissues (Tewksbury and Nabhan, 2001).

While there are more than ten different capsaicinoids in chili peppers, capsaicin is the most dominant and contributes the most to the pungency of chili peppers (Tewksbury and Nabhan, 2001; Barbero et al., 2006; Barbero et al., 2008; Naves et al., 2019). To broaden understanding of capsaicin levels in superhot chili, milder chili, and sweet peppers, the objective of this study was to quantify capsaicin levels in various chili peppers and determine the ranking of pungency based on capsaicin concentrations. We hypothesize that dry extraction at 60 °C increases yield by more than twofold compared to wet extraction, and that Habanero peppers will have higher yields than Thai and Serrano varieties due to greater vesicle density. We used a range of chili varieties, including Jalapeño, Habanero, Poblano, Serrano, Anaheim, Bell pepper, and Bird’s eye chili (Thai chili), to enhance understanding of capsaicin levels in peppers and to develop a relative “hotness” ranking among the peppers.

## 2 Materials and methods

### 2.1 Selection and procurement of chili peppers

We procured ripe chili peppers from the local market in three different batches at different time intervals. These chili peppers were obtained in California, USA, during the winter season of 2025 and analyzed within 48 hours of procurement. Prior to analysis, peppers were stored at 4 °C. The majority of these peppers sold in California come from various sources, including both local California farms and farms from other regions such as New Mexico. The chili peppers tested in this study included: 1) Shishito Peppers (SHP); 2) Anaheim Pepper (ANP); 3) Poblano Pepper (POP); 4) Thai Chili Pepper (THC); 5) Jalapeño Pepper (JAP); 6) Serrano Pepper (SEP); 7) Habanero Pepper (HAP); and 8) Red Bell Pepper (RBP). We obtained 0.5 kg of each chili pepper type. In each batch, approximately 350 g of each chili type was blended. The green Thai chili peppers were unripe, whereas orange Habanero peppers were ripe. Green Anaheim peppers were ripe, while green Poblano peppers were mature and not fully ripe. Both green Shishito peppers and Jalapeño peppers were ripe, whereas green Serrano peppers were unripe.

### 2.2 Chemicals and reagents

We used HPLC-grade water, formic acid, and acetonitrile to prepare the mobile phase of HPLC. The purity level of solvents (acetonitrile, ethanol, and acetone) used in this study was ≥99.9% (analytical grade). Fresh chili peppers were washed using ultrapure water obtained from a Milli-Q system (Millipore, Burlington, MA, USA). All solvents were purchased from Sigma-Aldrich (Sigma-Aldrich, Inc., St. Louis, MO, USA). To develop the HPLC-based method for capsaicin detection, we purchased an analytical-grade capsaicin standard (NIST Capsaicin) (molecular formula: C_18_H_27_NO_3_; IUPAC name: (*E*)-*N*-(4-Hydroxy-3-methoxybenzyl)-8-methylnon-6-enamide (CAS Number: 404-86-4; Molecular Weight 305.41; Sigma-Aldrich, Inc., St. Louis, MO, USA). HPLC vials, caps, conical tubes, and centrifuge tubes were purchased from Sigma-Aldrich (Sigma-Aldrich, Inc., St. Louis, MO, USA).

### 2.3 Sample preparation, extraction, and cleanup

**Figure 2:**
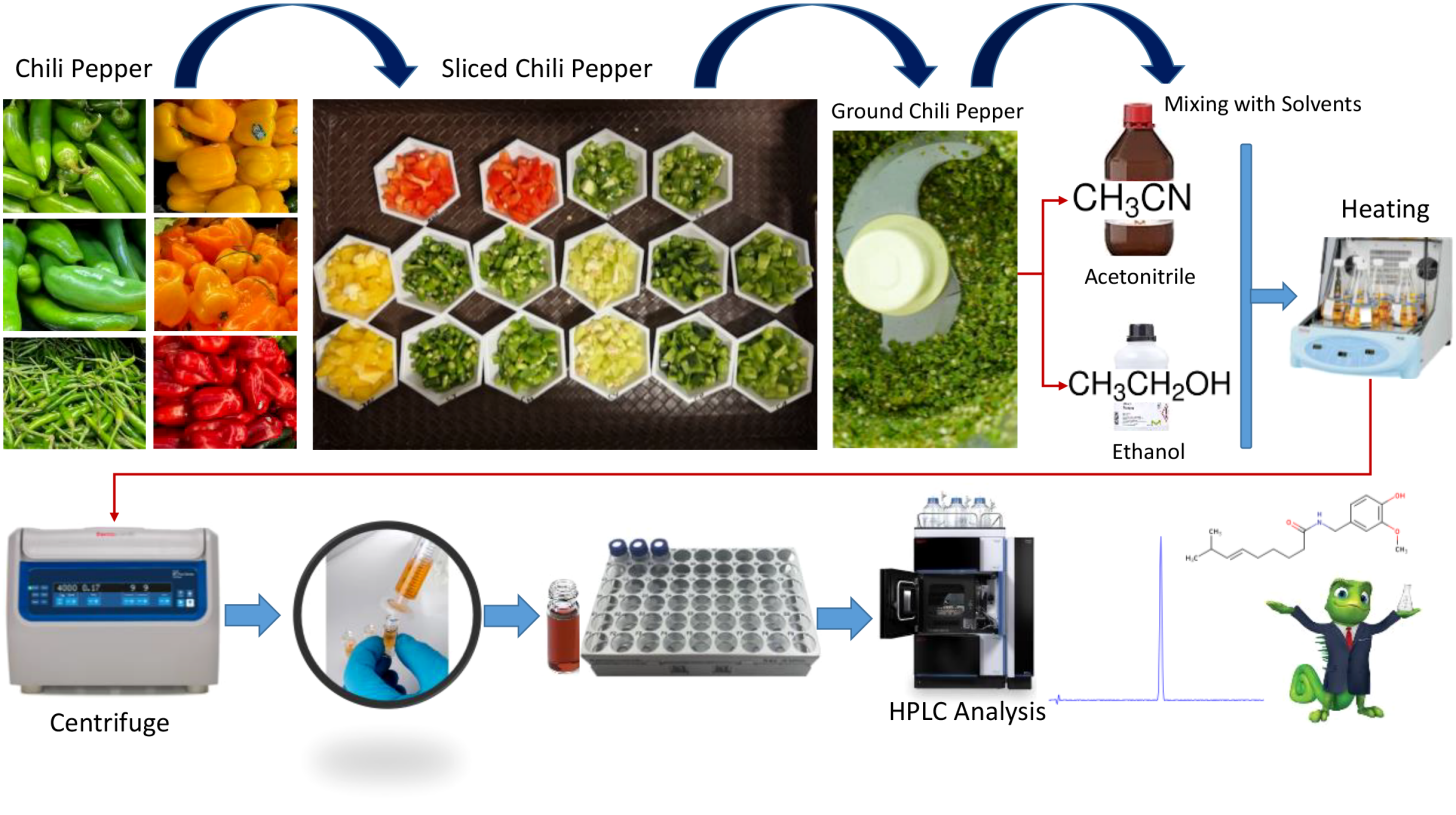
Overall sample preparation workflow. Top (left to right): fresh chilies, sliced chilies, ground chilies, extraction solvents, and incubator. Bottom (left to right): sample cleaning by centrifugation, filtration, and HPLC.

To detect capsaicin in peppers, a standardized sample processing method, including washing, cutting, grinding, mixing, heating, and extraction, was developed. The overall workflow is shown in **Figure 2**, which outlines the sample-processing steps. Uniform slicing of the peppers was needed before grinding. Subsequently, a blender (Blendjet Inc, Benicia, CA, USA) was used to convert sliced peppers into pepper purées. We used two methods to extract capsaicin from various chili peppers: 1) wet extraction, which involves extracting capsaicin from peppers without drying them; and 2) dry extraction, which requires drying ground chili peppers at 60 °C for two h. These two different extraction methods were applied to all chili peppers. While the ideal temperature for capsaicin extraction depends on the method of extraction, a range of 50-60 °C is commonly used during Soxhlet-based extraction to ensure thermal stability (Barbero et al., 2006; Barbero et al., 2008). In other methods, such as microwave-assisted extraction and pressurized liquid extraction, temperatures of 125 °C and 80 °C, respectively, have been reported (Barbero et al., 2006; Barbero et al., 2008; Bajer et al., 2015). Studies suggest that drying at 60 °C for 2–4 h with gentle mixing is favorable in order to avoid thermal degradation of capsaicinoids (Korkutata and Kavaz, 2015; Zhou et al., 2016; Yap et al., 2022; Krzykowski et al., 2024).

During the extraction process, acetonitrile was mixed with ground chili peppers, and the mixture was kept at 60 °C for 2 h under gentle shaking conditions at 150 RPM/0.11 RCF (×g) (Thermo Scientific MaxQ 4450 Digital Orbital Incubator Shaker). At the end of extraction, the color (green, yellow, and red) of the mixture varied depending on the type of chili peppers. This mixture contained numerous particles, and particle separation was needed prior to HPLC analysis. We centrifuged the mixture at 2,500 RCF (×g) for 5 min. The supernatant was transferred to 2 mL centrifuge tubes. Subsequently, these tubes were centrifuged in a micro centrifuge (Thermo Sorvall Legend Micro centrifuge) at 2,900 RCF (×g) for 6 min. After centrifuging, samples were filtered through Whatman UNIFLO 25 Syringe Filters (0.2 µm) and transferred to HPLC vials for further analysis.

### 2.4 High-performance liquid chromatography conditions

Capsaicin in peppers was tested using the Thermo Vanquish Core HPLC System (Thermo Scientific, Waltham, MA, USA). This HPLC system included a diode array detector (P/N: VC-D11-A; S/N: 8323898), quaternary pump (P/N: VC-P20-A; S/N: 8323772), column compartment (P/N: VC-C10-A; S/N: 6507213), and split sampler (P/N: VC-A12-A; S/N 8323883). The system was operated and controlled using Thermo Chromeleon 7 Single Edition with Spectral License (3D/MS) software. During analysis, HPLC separation was performed using a SiELC column (Newcrom R1 HPLC column, 100 × 3.2 mm × 3µm) (SIELC Technologies, Wheeling, IL, USA). The temperature of the column compartment was maintained at 30 °C during sample analysis. The HPLC flow rate was set to 0.4 mL/min under isocratic conditions. HPLC mobile phase included: A) 0.5% formic acid mixed with HPLC-grade water (30%); and B) 100% acetonitrile (70%) [without creating a gradient]. Before running samples, column conditioning was performed by passing acetonitrile (70%) and water (30%) for 1 h at 0.8 mL/min. Capsaicin peaks were detected at three wavelengths (220 nm, 236 nm, and 280 nm) using a UV-Vis diode array detector. The purpose of using three wavelengths was to compare capsaicin responses on these three wavelengths and select the wavelength with the highest signal. Three blank samples and three spiked blanks were tested to evaluate matrix effects. The autosampler temperature was set to 4 °C during the test, and the injection volume was 10 µL.

### 2.5 Sensitivity and linearity

**Figure 3:**
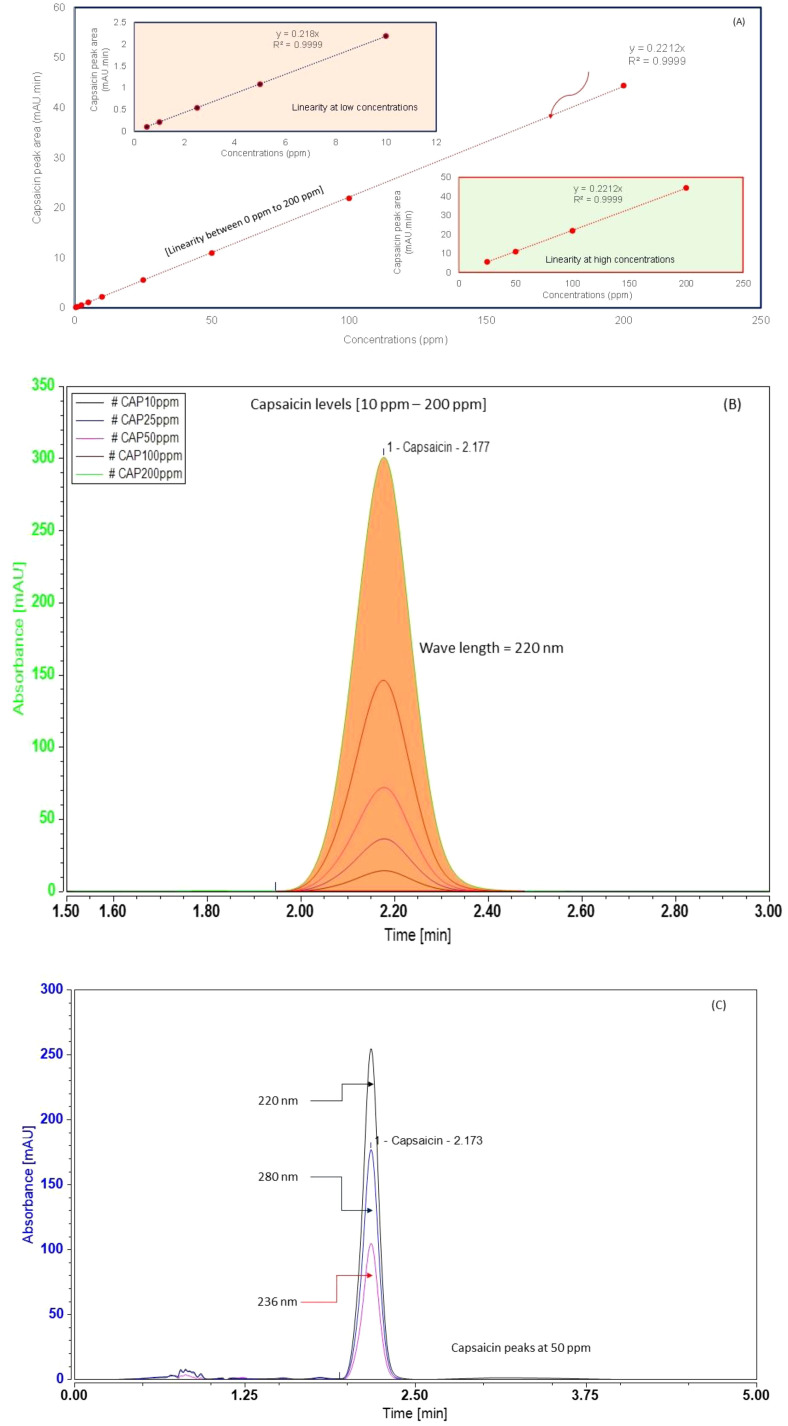
Chromatograms of capsaicin at various concentration levels: **(A)** calibration concentrations linearity [at 220 nm]; **(B)** capsaicin peaks at different concentrations (220 nm); and **(C)** capsaicin peaks at different wavelengths at 50 ppm. The linearity line includes both low concentrations (0.5 ppm to 10 ppm) and high concentrations of capsaicin (25 ppm to 200 ppm).

Prior to sample analysis, we estimated the sensitivity and linearity of the HPLC method. Sensitivity was assessed using the signal-to-noise ratio (SNR). The limit of detection (LOD) was defined by an SNR ratio of 3:1. The limit of quantification (LOQ) was defined by an SNR ratio of 10:1. To determine linearity, we created a calibration curve by plotting diode array detector response versus concentration of a series of standards (0.025 ppm, 0.05 ppm, 0.1 ppm, 0.5 ppm, 1 ppm, 2.5 ppm, 5 ppm, 20 ppm, 25 ppm, 50 ppm, 100 ppm, and 200 ppm), and calculated the correlation coefficient (*r^2^*) using linear regression. During calibration, we obtained an *r^2^* value of 0.99. Known concentrations of capsaicin were plotted against capsaicin peak areas, and the resulting *r^2^* value is shown in **Figure 3**.

### 2.6 Accuracy, repeatability, and precision

The accuracy and precision of the HPLC method were evaluated through recovery and reproducibility studies using known concentrations of capsaicin in the aqueous phase. Recovery was calculated as the ratio of detected to added concentrations. Reproducibility was assessed by performing six replicate injections and repeating analyses on the same samples across different days, with samples stored at 4 °C between tests. Reproducibility ranged from 2% to 4%. Instrument precision was determined by evaluating the repeatability of the measurements under identical conditions, while accuracy was assessed by comparing measured concentrations with actual concentrations.

### 2.7 Statistical analysis

To perform statistical analysis, we used GraphPad Prism 5 (GraphPad Software, LLC, Boston, MA, USA) and Microsoft Excel 2016 (Microsoft Corporation, Redmond, WA, USA). To visualize and evaluate capsaicin chromatograms, we used Thermo Scientific™ Chromeleon™ Chromatography Data System (CDS). For descriptive statistic calculations (average, standard deviation, error, range, comparison, and linear regression), we used Microsoft Excel 2016. To compare Scoville Heat Units (SHU) among pepper types, capsaicin mg/kg was converted to SHU as described previously (Canto-Flick et al., 2008b; Douventzidis and Landquist, 2022) (SHU = capsaicin mg/kg × 16). Pairwise ANOVA using Tukey HSD test (P< 0.05) was performed to determine the significant differences among the seven pepper cultivars.

## 3 Results and discussion

### 3.1 Linearity, sensitivity, precision, and repeatability

Chromatograms of capsaicin are shown in **Figures 3A, B**. The identification of peaks was performed at three wavelengths (λ_max_ = 220 nm, 280 nm, and 236 nm). Capsaicin was detectable at all three wavelengths; however, the highest signals (peak area) were obtained at 220 nm (**Figure 3C**). The overlapping peaks of capsaicin at various concentrations (**Figure 3B**) indicate that the retention time (*R_t_*) of the peaks was consistent (*R_t_* – 2.173 min). The detector wavelength of 220 nm yielded improved signals and peak characteristics (i.e., greater peak areas and elevated peak heights) for a given capsaicin concentration. The peak areas for various capsaicin concentrations (0.5 ppm, 1 ppm, 2.5 ppm, 5 ppm, 10 ppm, 25 ppm, 50 ppm, 100 ppm, and 200 ppm) are presented in **Figure 3** (additional information is shown in **Supplementary Table 1**, **Supplementary Figure 1**). The linearity of the curve is also shown in **Figure 3**. The linearity test resulted in an *R^2^* value of 0.9999. The LOD and LOQ values were 0.5 and 0.75 ppm, respectively. The relative standard deviation (RSD) for inter-day analysis (n = 6) was < 5%, and repeatability RSD was < 6% (n = 6). The recovery value ranged between 90% and 100%. The precision of the capsaicin method was 2-5%, and the accuracy was within 5%.

### 3.2 Impacts of solvents on capsaicin extractions and moisture content of chili peppers

**Figure 4:**
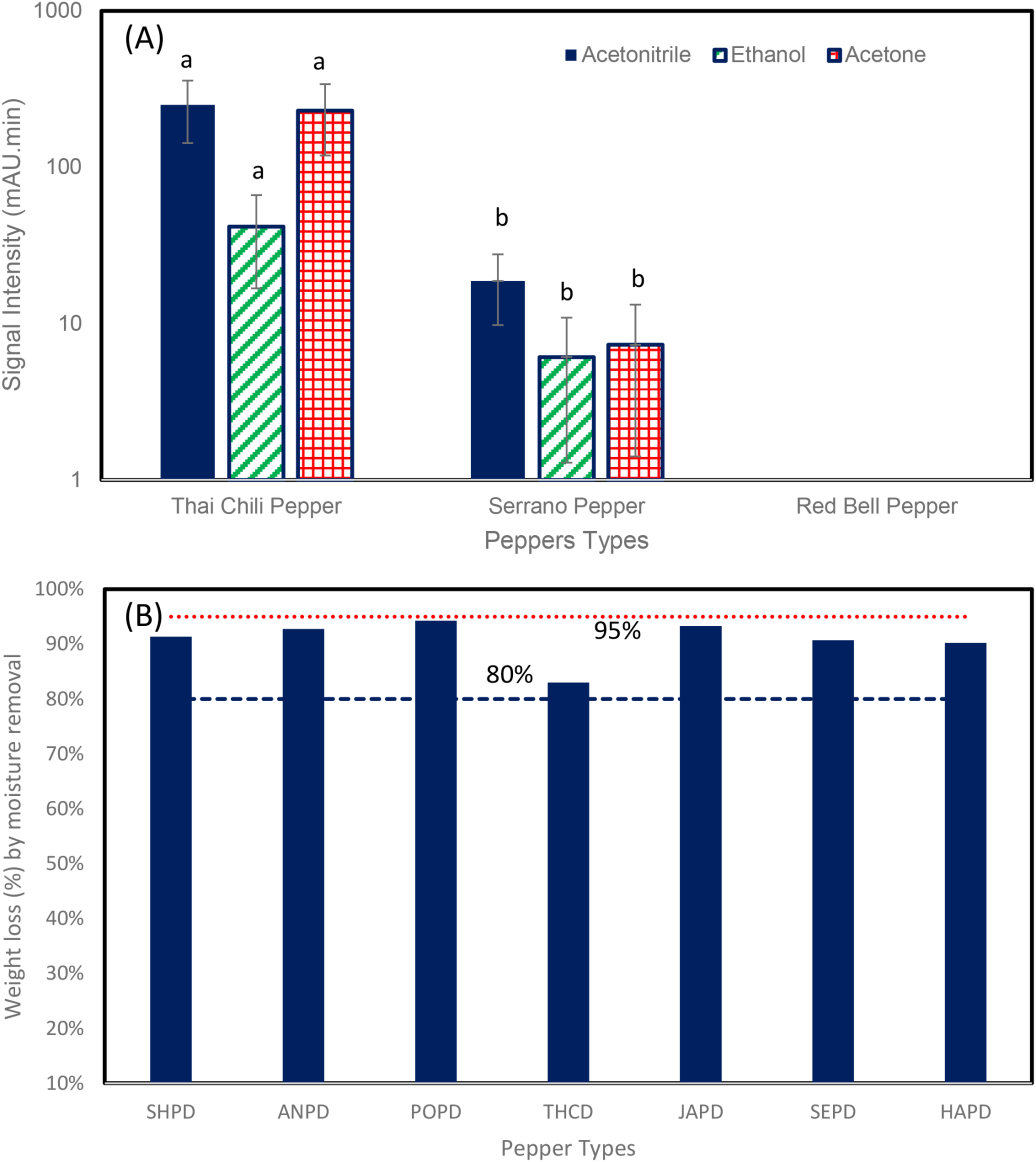
**(A)** Effects of different solvents (acetonitrile, ethanol, and acetone) on capsaicin extraction from fresh ground chili [bar chart with similar lowercase letters are not statistically different, p ≤ 0.05 (Tukey's test)]. **(B)** Weight loss of different types of chili during drying at 60 °C for 24–30 (h) Drying was conducted prior to capsaicin extraction. Chili types included SHPD, Shishito Peeper Dried; ANPD, Anaheim Pepper Dried; POD, Poblano Pepper Dried; THCD, Thai Chili Pepper Dried; JAPD, Jalapeno Pepper Dried; SEPD, Serrano Pepper Dried; HAPD, Habanero Pepper Dried. Each experiment was performed in triplicate.

In order to test capsaicin extraction from chili peppers using acetonitrile, ethanol, and acetone (**Figure 4**), we used three types of chili peppers (Thai chili pepper, Serrano pepper, and Red Bell pepper). Fresh ground chili peppers (4 g) were mixed with 8mL of solvent (i.e., the solvent was two times the chili pepper weight, that is, solvent-to-ground-pepper-weight ratio was 2) and were subjected to mixing (150 RPM/0.11 RCF (×g)) and heating at 60 °C for 2 h. There was no sonication process involved in this study. The three solvents were acetonitrile, ethanol, and acetone (**Figure 4**). The peak areas of capsaicin in these three solvents (acetonitrile, ethanol, and acetone) were estimated at three wavelengths (220 nm, 236 nm, and 280 nm). Subsequently, average peak areas for each pepper were calculated using the individual peak areas obtained from all three wavelengths. The signal intensity of capsaicin in these three solvents is shown in **Figure 4**. The results showed that acetonitrile produced the highest capsaicin signals.

In addition, results showed that capsaicin levels in Thai chili pepper and Serrano pepper were high; however, no capsaicin signals were detected in Red Bell peppers. Red Bell pepper is considered a type of sweet pepper and does not produce capsaicin (Korkutata and Kavaz, 2015; Zhou et al., 2016; Yap et al., 2022; Krzykowski et al., 2024). Regarding moisture content of peppers, various chili peppers were dried for 24–30 h at 60 °C to remove water content. Results (**Figure 4B**) show that the moisture content of chili peppers ranged between 82% and 94%, depending on pepper type. The highest moisture content (94.2%) was found in Poblano pepper, while the lowest (82.9%) was observed in Thai chili peppers.

### 3.3 Comparative extraction of capsaicin levels in wet and dried peppers

**Figure 5:**
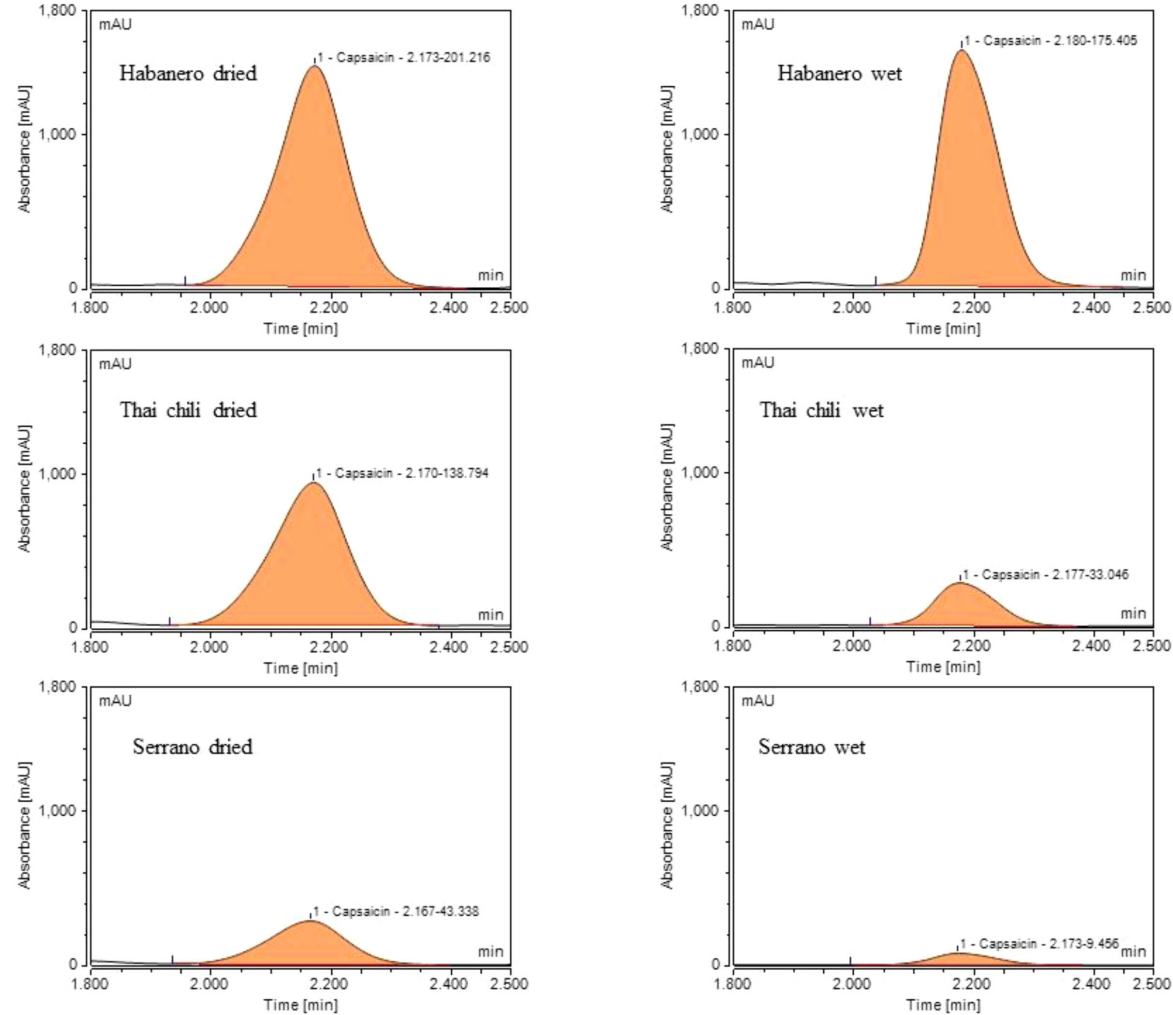
Chromatograms of capsaicin in chili peppers (Habanero, Thai chili, and Serrano peppers). Left-side chromatograms show capsaicin in solvent extracts following dry extraction, while right-side chromatograms show capsaicin in solvent extracts from fresh ground chili peppers (wet extraction, no drying prior to extraction). Each chromatogram shows retention time followed by peak area (i.e., retention time-peak area).

To evaluate the effect of moisture on capsaicin peaks and extraction, we mixed fresh ground chili with acetonitrile. For comparison, dried samples were prepared by mixing desiccated ground chili (i.e., dried at 60 °C) with acetonitrile. The solvent-to-pepper ratio (weight basis) was 2:1 for both wet and dried peppers. Chromatograms (**Figure 5**) show that the drying process enhanced capsaicin extraction. Chromatograms of capsaicin from three different types of chili (Habanero, Thai chili, and Serrano peppers) show that the intensity of capsaicin peaks in dried chili peppers was higher, and peak areas were increased. For example, the peak area of dried Habanero was 35.5% higher than that of Habanero wet extracts. In Thai chili peppers, capsaicin peak areas of dried pepper extracts were 43.8% higher than those of wet extracts. In Serrano pepper, capsaicin peaks were considerably smaller than those of Habanero and Thai chili peppers. However, the peak area of capsaicin in dried Serrano extract was 98.6% higher than that of the wet extract. Overall, comparison of capsaicin peaks between dry extraction and wet extraction indicates that drying peppers prior to mixing with solvents improves the extraction of capsaicin across all chili types tested in this study.

### 3.4 Profiling of capsaicin levels in various chili peppers

**Figure 6:**
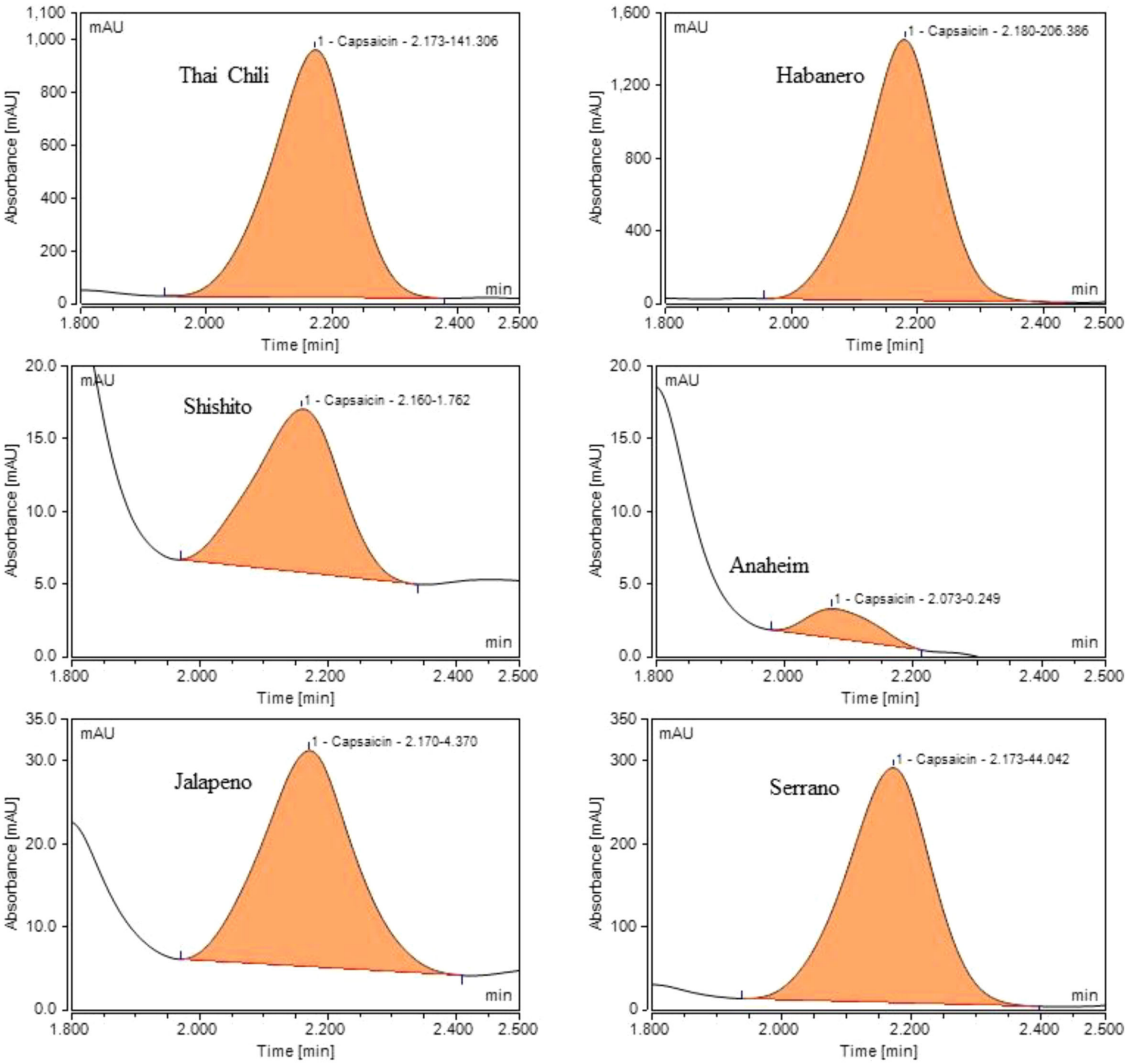
Capsaicin chromatograms of six chili peppers (post-dry extraction) (Thai chili, Habanero, Shishito, Anaheim, Jalapeño, and Serrano peppers) [Note: scales of each chromatogram vary to enhance chromatogram visualization. Capsaicin peaks of Anaheim, Shishito, and Jalapeño were substantially smaller than the capsaicin peaks of Thai chili, Habanero, and Serrano. Each chromatogram shows retention time followed by peak area (i.e., retention time-peak area).

To develop a capsaicin profile for seven chili pepper cultivars and quantify capsaicin levels, we used a dry extraction method. First, the moisture of fresh ground chili peppers was removed by heating at 60 °C for 30–40 h, and the weight loss (%) of peppers was recorded (**Figure 4**). After drying, acetonitrile was mixed with dried chili peppers (solvent-to-pepper ratio of 2:1), and the mixture was heated for 2 h at 60 °C with gentle mixing and sealed to avoid solvent evaporation. The capsaicin peaks in Thai chili, Habanero, Shishito, Anaheim, Jalapeño, and Serrano peppers are shown in **Figure 6**. Chromatograms indicate that the capsaicin peak was the highest in Habanero, followed by Thai chili, Serrano, Jalapeño, Shishito, and Anaheim Peppers. The capsaicin peak area for Habanero was 1.46 times greater than that of Thai chili and 4.68 times greater than that of Serrano.

**Figure 7:**
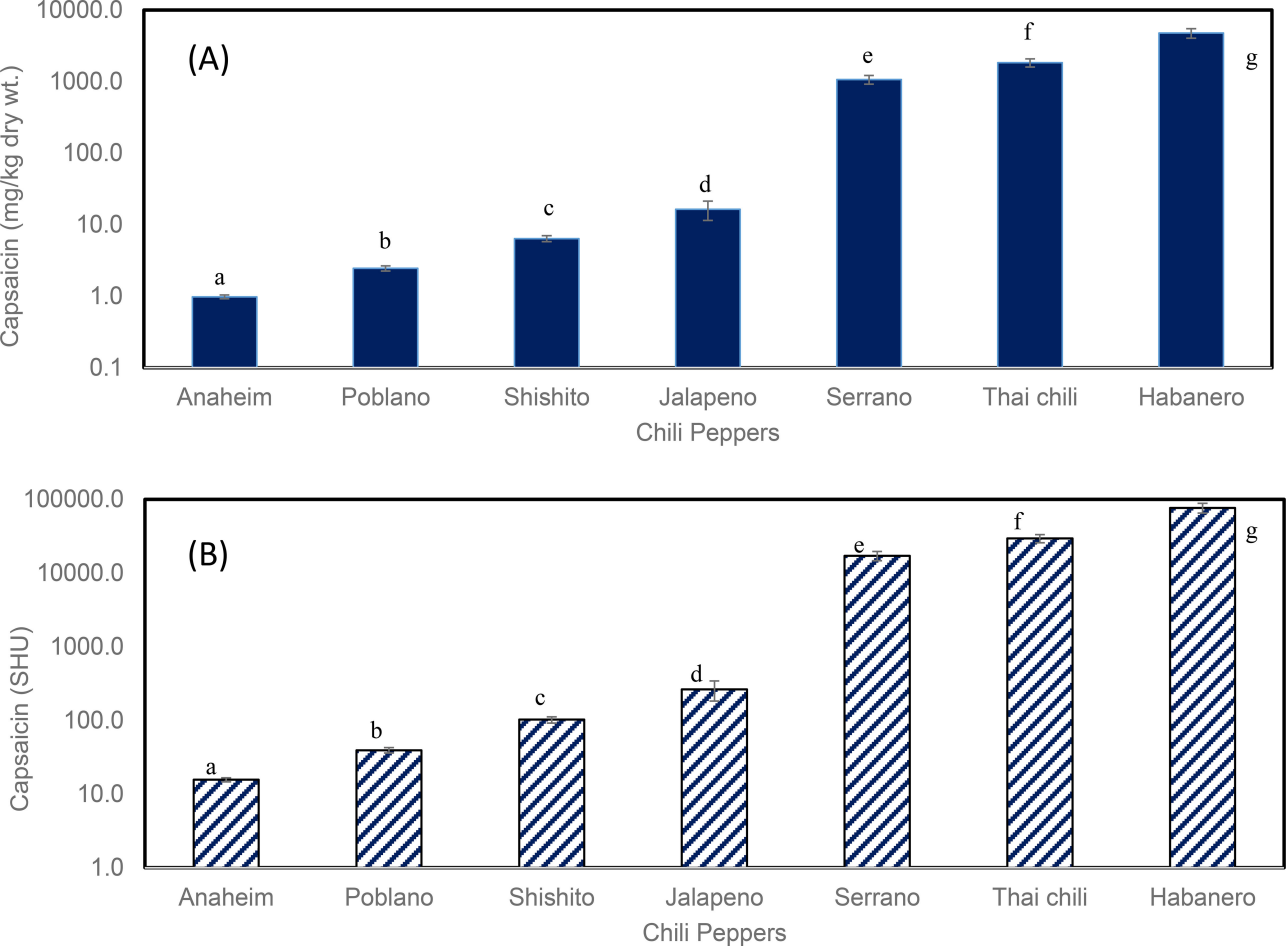
Capsaicin content **(A)** mg/kg dry wt, mean ± SD, n = 3; **(B)** SHU, mean ± SD, n = 3; SHU = capsaicin mg/kg × 16] in seven pepper cultivars. Different letters indicate significant differences (p< 0.05; Tukey’s test).

The capsaicin peak area in Habanero was 601 times greater than that in Jalapeño pepper. **Table 1** shows capsaicin concentrations in seven pepper cultivars. In addition, the capsaicin ratio between wet and dried peppers is shown in **Table 1**. Using capsaicin peak areas for each pepper, dilution factors, and biomass weights, we estimated capsaicin quantity (mg/kg of dry weight) for each pepper type. Capsaicin concentrations for various peppers are shown in **Figure 7**.

**Table 1:** Capsaicin concentrations in seven cultivars post-dry extraction (numbers followed by different lowercase letters are statistically different, p ≤ 0.05 (Tukey's test);each experiment was performed in triplicate).

Chili pepper types	Mean capsaicin (mg/kg dry wt)
Anaheim	0.98 (± 0.06)^a^
Poblano	2.46 (± 0.21) ^b^
Shishito	6.38 (± 62) ^c^
Jalapeño	16.44 (± 4.97) ^d^
Serrano	1069.32 (± 152.22) ^e^
Thai chili	1844.84 (± 239.40) ^f^
Habanero	4777.56 (± 720.89) ^g^
Capsaicin ratio among extraction procedure	
	Capsaicin Ratio (wet/dry) [mg/kg]
Jalapeño	0.08
Serrano	0.09
Thai chili	0.17
Habanero	0.16

Means with different superscript letters in the right column differ significantly (P < 0.05).

These results showed that capsaicin content was highest in Habanero (4777.56 ± 720.89 mg/kg dry wt), while the lowest capsaicin content was found in Anaheim (0.98 ± 0.06 mg/kg of dry wt). The ranking of peppers in terms of capsaicin quantity (from higher to lower) was: Habanero (4,777.56 ± 720.89 mg/kg dry wt), Thai chili pepper (1,844.84 ± 239.4 mg/kg dry wt), Serrano (1069.32 ± 152.22 mg/kg dry wt), Jalapeño (16.44 ± 4.97 mg/kg dry wt), Shishito (6.34 ± 0.62 mg/kg dry wt), Poblano (2.46 ± 0.21 mg/kg dry wt), and Anaheim (0.98 ± 0.06 mg/kg dry wt). Capsaicin in Habanero was 158% greater than in Thai chili, 357% greater than in Serrano, and 28,964% greater than in Jalapeño peppers. In general, capsaicin levels in Shishito, Poblano, and Anaheim were low (**Figure 7**). In Red Bell pepper, regardless of the method used (dry extraction or wet extraction), capsaicin was not detectable; therefore, these data are not plotted in **Figures 6**, **7**.

Regarding the understanding of capsaicin in peppers, a previous study (Korkutata and Kavaz, 2015) compared the morphological and histological characteristics of the interlocular septa of Jalapeño and Bell peppers using scanning and transmission electron micrographs. Results showed little histological differences of the interlocular septa, and the authors concluded that capsaicin is synthesized in the glandular areas of the interlocular septum of Jalapeño peppers. The cross walls of pungent peppers contain gland cells with yellow, orange, and green pigments, and the increased secretion from these cells results in a greater amount of capsaicin (Aza-González et al., 2011; Korkutata and Kavaz, 2015). Peppers such as Habanero and Thai chili are recognized among the most pungent peppers (Zhou et al., 2016), and previous studies suggest that the abundant transcripts of additional genes are responsible for the high level of pungency of Habanero peppers (Aza-González et al., 2011). Traditionally, the heat of chili peppers is measured by the Scoville Heat Unit (SHU) scale. For example, the heat levels of orange Habanero (grown in Las Cruces, New Mexico, USA) are high (SHU of 357,729). Other extremely hot peppers, such as Bhut Jolokia (a chili pepper from Assam, India), showed a very high heat value of 1,001,304 SHU (Bosland and Baral, 2007). Morphological analyses of these chili peppers indicate that the quantity of capsaicinoid secretion from vesicles regulates pepper heat (Bosland et al., 2015).

**Figure 8:**
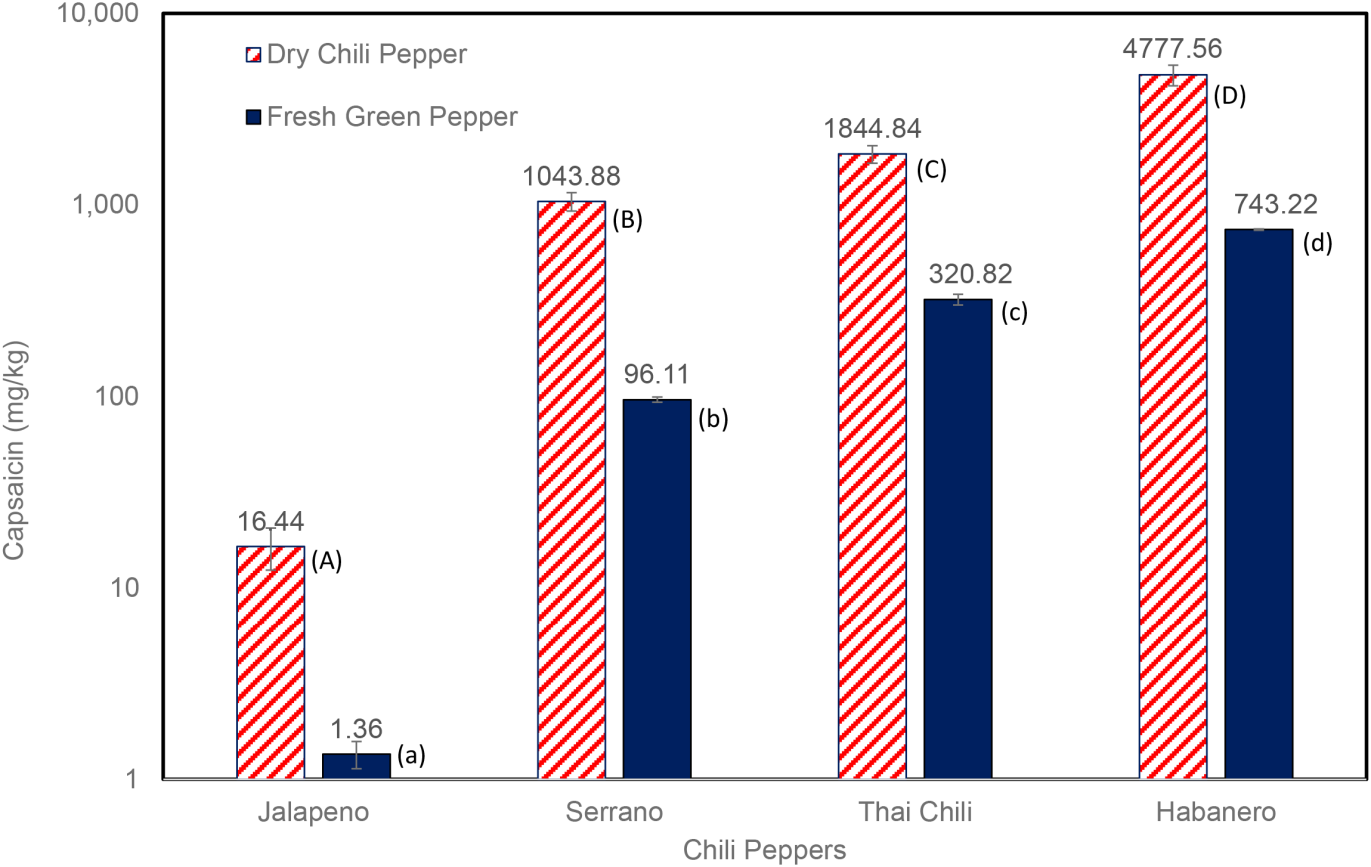
Capsaicin content in pepper cultivars (post-dry extraction and wet extraction). Different uppercase and lowercase letters indicate significant differences among groups (p< 0.05; Tukey’s test). Error bars show the standard deviation (±) of three replicates.

To quantify capsaicin yield during wet and dry extraction, we estimated capsaicin concentrations on a mass basis (mg/kg). Capsaicin yields during wet and dry extractions for Jalapeño, Serrano, Thai chili, and Habanero peppers are shown in **Figure 8**. The capsaicin yield in dried Habanero was 542% greater than that of wet Habanero. In Thai chili, the capsaicin yield in dried samples was 475% greater than in wet extraction. In the Serrano pepper, the capsaicin yield in dry extraction was 986% higher than in wet extraction. Similarly, the drying process increased capsaicin yield in Jalapeño by 1,108.8% compared to wet extraction. Elevated Habanero levels align with ectopic vesicles in the pericarp (Bosland et al., 2015), and dry extraction may have concentrated capsaicinoids by removing water interference. These results indicate that capsaicin yields in dried peppers are substantially greater than in wet peppers, and that moisture reduces capsaicin extraction efficiency across all pepper types tested.

This study found that Habanero and Thai chili peppers have substantially higher capsaicin levels than other peppers, likely due to a combination of environmental factors and genetic predisposition (Rowland et al., 1983; Madhavi Reddy et al., 2016). Elevated levels of capsaicin in these peppers indicate abundant biosynthesis and accumulation in pepper tissues (Suzuki et al., 1981; Welch et al., 2014; Azlan et al., 2022). The SHU of Habanero peppers ranges between 100,000 and 350,000 SHU (Stewart et al., 2007; Canto-Flick et al., 2008a), while Thai chili pungency ranges from 45,000 to 80,000 SHU (Canto-Flick et al., 2008a; Palma-Orozco et al., 2021). In contrast, Bell Peppers have 0 SHU (Bosland et al., 2015). In terms of the accumulation of capsaicin in various peppers, genetic factors are known to influence capsaicinoid content in peppers, and, to a great degree, the interaction between genotype and environment also influences capsaicinoid content (Palma-Orozco et al., 2021). The high level of pungency of Habanero and Thai chili peppers is therefore closely associated with elevated levels of capsaicin.

Overall, capsaicinoids are unique to pepper fruits and are a group of compounds responsible for chili pepper’s pungency. In most cases, capsaicinoid biosynthesis occurs in placental cells, and the resulting capsaicinoids are stored within blisters on the placental surface (Bosland and Votava, 2012; Bosland and Baral, 2007; Aza-González et al., 2011). In general, capsaicinoids from chili peppers provide numerous vitamins and minerals beneficial to human health (Mejia et al., 1988; Matus et al., 1991; Marín et al., 2004). Capsaicinoids possess a series of beneficial physiological and pharmacological effects, such as anticancer, anti-inflammatory, antioxidant, and anti-obesity properties. Due to their wide applications in food and pharmaceuticals, global pepper production has increased by 40% in the past decade (Bosland and Votava, 2012; Buettner, 2014; Zhou et al., 2016). These unique characteristics of peppers have motivated extensive research into capsaicinoids in various chili peppers and their biological functions (Fei et al., 2025; Wang et al., 2025; Kraikruan et al., 2008b; Othman et al., 2011; Stoica et al., 2016; Martins et al., 2017; Ksh et al., 2023; Beyyavaş and Aslanoglu, 2025; Gyalai et al., 2025). Because capsaicinoids, such as capsaicin levels, change substantially among various pepper types (Zhang et al., 2025; Lim et al., 2025; Zhao et al., 2025), there is a further need for comparative studies that conduct extraction and test capsaicin levels in various types of peppers using uniform methods (Yu et al., 2014; Zhao et al., 2025).

In terms of heat content in chili peppers, HPLC-based estimates range from 0 (no heat, as observed in bell peppers) to >1,000,000 (as observed in superhot cultivars such as *Capsicum chinense*, e.g., habanero-type peppers). Genes such as Serine Acetyltransferase 2 (SAT2) and Histone-Lysine N-Methyltransferases (HKMTs) have been shown to influence the biosynthesis of capsaicinoids (Khan et al., 2025). Another study conducted a genome-wide association study (GWAS) to elucidate the genetic architecture of pepper metabolomes, thereby improving our understanding of the biosynthesis of species-specific capsaicinoids (von Steimker et al., 2024). A study on the evolution of pungency in *Capsicum* used two telomere−to−telomere, gap−free genomes—those of *C. annuum* and its wild, non−pungent relative *C. rhomboideum*—and revealed that conserved, placenta−specific chromatin regions enable tissue−specific regulation of biosynthetic genes and capsaicinoid accumulation (Kim et al., 2014, Chen et al., 2024). Reactions such as the conversion of vanillin to vanillylamine are unique steps in the biosynthesis of pungent capsaicinoids, which occur exclusively in chili peppers (Kusaka et al., 2024). Advanced tools and capsaicinomics-based methods can describe various pathways related to sulfur metabolism and hormone signaling, which regulate heat content in peppers. Understanding capsaicin genomics through the integration of phenotypic data with genomic information obtained from sequencing provides insights into complex polygenic architectures underlying pepper fruit quality traits and capsaicinoid levels. The integration of pangenomics and multi−omics approaches not only deepens our understanding of natural compound biosynthesis but also offers new opportunities for improving Capsicum cultivars, in addition to enhancing potential application in breeding (Khan et al., 2025; Vega-Muñoz et al., 2025).

In summary, an advanced understanding of this important molecule (Caterina et al., 1997; Vega-Muñoz et al., 2025) in widely cultivated peppers is crucial for both producers and consumers. Therefore, this research aimed to improve existing knowledge of capsaicin in various peppers, including capsaicin extraction methods and HPLC-based detection. Both wet and dry extraction methods were explored to determine the impact of moisture on capsaicin extraction yield. The capsaicin extraction process is crucial to the pharmaceutical industry for isolating, purifying, and concentrating capsaicinoids. In this study, we used reversed-phase HPLC with a UV-Vis detector. Results showed that dry-pepper extractions were better than wet-pepper extractions (dry extraction yielded 542%-1,110% more capsaicin than wet extractions). This finding highlights the significant impacts of drying on capsaicin extraction efficiency. Comparative analysis showed that hot chili varieties, such as Thai chili, Habanero, and Serrano, have substantially higher capsaicin levels than milder varieties, such as Jalapeño, Shishito, Poblano, and Anaheim. Based on capsaicin content, peppers ranked as follows (from highest to lowest): Habanero > Thai chili > Serrano > Jalapeño > Shishito > Poblano> Anaheim. HPLC-based quantification offers an objective and reproducible alternative to the Scoville Heat Units (SHU) scale for assessing the spiciness of peppers. Existing Scoville ratings for these chili peppers vary widely: Anaheim — 3,400 SHU; Habanero — 36,000–502,000 SHU; Thai — 30,000–50,000 SHU; Serrano — 25,000–43,000 SHU; Jalapeño — 228–62,900 SHU; Shishito — 50–200 SHU; Poblano — 1,000–1,500 SHU; and Red bell — 0–1,000 SHU (Canto-Flick et al., 2008b; Douventzidis and Landquist, 2022). Additional improvements in ratings can assist in characterizing these peppers based on quantitative values of capsaicinoids. We anticipate that the information provided here will be valuable to scientists and processors involved in capsaicin extraction and detection, in addition to those exploring the use of peppers for food applications and human health.

## 4 Conclusions

Peppers play a crucial role in food systems by enhancing flavor and providing health benefits. However, the underlying principles and mechanisms governing this flavor remain unclear to many, including growers. While capsaicin extraction and use of HPLC have been investigated previously, this study provides unique information in terms of quantification of capsaicin levels in seven chili pepper cultivars using optimized HPLC. The results establish a clear ranking of chili peppers based on capsaicin concentrations. Our results and findings provide a unifying understanding that explains previously disparate experimental tests and observations regarding capsaicin levels in various peppers, including Thai chili, Habanero, Serrano, Jalapeño, Shishito, Poblano, and Anaheim. This work is useful for the development of advanced methods for rapidly assessing pungency levels, which helps to implement future technologies in agriculture for the quantitative assessment of spiciness in various peppers.

## Data Availability

The datasets presented in this study can be found in online repositories. The names of the repository/repositories and accession number(s) can be found in the article/**Supplementary Material**.
