# Supplementary material for: Quantitative profiling of capsaicin content in seven chili pepper cultivars using optimized HPLC
Source: Front Plant Sci. 2026 May 7;17:1749488. doi: 10.3389/fpls.2026.1749488 (PMC13190488; doi:10.3389/fpls.2026.1749488)
Supplement: Supplementary file 4 [file Table1.docx]

**Figure S1:** Capsaicin calibration curves and associated data points.

| **Concentrations (ppm)** | **Areas (mAU.min)** |
| --- | --- |
| 0.5 | 0.1024 |
| 1 | 0.213 |
| 2.5 | 0.5385 |
| 5 | 1.0811 |
| 10 | 2.1874 |
| 25 | 5.5448 |
| 50 | 10.9355 |
| 100 | 21.87 |
| 200 | 44.3883 |
