# Supplementary material for: Quantitative profiling of capsaicin content in seven chili pepper cultivars using optimized HPLC
Source: Front Plant Sci. 2026 May 7;17:1749488. doi: 10.3389/fpls.2026.1749488 (PMC13190488; doi:10.3389/fpls.2026.1749488)
Supplement: Supplementary file 5 [file Table2.docx]

**Table S1: Supplemental information for capsaicin detection.**

| **Items** | | **Descriptions** |
| --- | --- | --- |
| Solvents | Acetonitrile, ethanol, acetone | |
| Wave lengths | 220 nm, 236 nm, 280 nm | |
| HPLC types | Thermo Vanquish Core | |
| Detector type | UV-Vis | |
| Column Type | Newcrom R1 HPLC column, 100 × 3.2 mm × 3µm | |
| Column Temperature | 30 °C | |
| HPLC flow rate | 0.4 mL/min | |
| Mobile phases | A) 0.5% formic acid mixed with HPLC grade water (30%); and B) 100% acetonitrile (70%). | |
| Capsaicin peak retention time | 2.173 min at 220 nm | |
| Limit of detection (LOD) | 0.025 ppm | |
| Calibration standards | 0.025 ppm, 0.05 ppm, 0.1 ppm, 0.5 ppm, 1 ppm, 2.5 ppm, 5 ppm, 20 ppm, 25 ppm, 50 ppm, 100 ppm, and 200 ppm | |
